# Supplementary material for: Boundary curves of individual items in the distribution of total depressive symptom scores approximate an exponential pattern in a general population
Source: PeerJ. 2016 Oct 11;4:e2566. doi: 10.7717/peerj.2566 (PMC5068372; doi:10.7717/peerj.2566)
Supplement: Supplemental Information 1 — Empirical constants are given for linear fitting with Y = a*X + b, for quadratic fitting with Y = a*X2 + b*X + c, for exponential fitting with Y= a*eb*X. The coefficient of determination R2 values for exponential fits were higher than those for linear or quadratic fits for all boundary curves. [file peerj-04-2566-s001.docx]

| Boundary curve | Linear | | | Quadratic | | | | Exponential | | |
| --- | --- | --- | --- | --- | --- | --- | --- | --- | --- | --- |
|  | a | b | *R*^2^ | a | b | c | *R*^2^ | a | b | *R*^2^ |
| Item 5, score 0-1 | -23.1 | 799 | *0.36* | 1.7 | -110 | 1535 | *0.68* | 2055 | -0.22 | *0.99* |
| Item 5, score 1-2 | -33.4 | 1195 | *0.57* | 1.9 | -131 | 2023 | *0.88* | 4652 | -0.20 | *0.98* |
| Item 5, score 2-3 | -35.7 | 1305 | *0.64* | 1.8 | -128 | 2088 | *0.91* | 4681 | -0.17 | *0.97* |
| Item 6, score 0-1 | -24.5 | 844 | *0.37* | 1.9 | -117 | 1632 | *0.70* | 3658 | -0.29 | *0.99* |
| Item 6, score 1-2 | -33.8 | 1205 | *0.57* | 2.0 | -135 | 2062 | *0.89* | 6242 | -0.24 | *0.95* |
| Item 6, score 2-3 | -35.8 | 1300 | *0.63* | 1.9 | -130 | 2103 | *0.91* | 5364 | -0.19 | *0.95* |
| Item 7, score 0-1 | -20.4 | 703 | *0.31* | 1.6 | -100 | 1380 | *0.61* | 2058 | -0.25 | *0.97* |
| Item 7, score 1-2 | -33.2 | 1185 | *0.56* | 2.0 | -131 | 2021 | *0.88* | 4763 | -0.21 | *0.98* |
| Item 7, score 2-3 | -35.5 | 1291 | *0.63* | 1.9 | -129 | 2088 | *0.91* | 4057 | -0.17 | *0.98* |
| Item 9, score 0-1 | -23.2 | 810 | *0.39* | 1.6 | -105 | 1509 | *0.70* | 2634 | -0.22 | *0.97* |
| Item 9, score 1-2 | -33.1 | 1186 | *0.57* | 1.9 | -129 | 2001 | *0.88* | 4718 | -0.20 | *0.97* |
| Item 9, score 2-3 | -35.6 | 1300 | *0.64* | 1.9 | -128 | 2088 | *0.91* | 4220 | -0.17 | *0.96* |
| Item 10, score 0-1 | -32.5 | 1158 | *0.55* | 2.0 | -131 | 1993 | *0.87* | 4889 | -0.21 | *0.97* |
| Item 10, score 1-2 | -35.7 | 1296 | *0.63* | 1.9 | -130 | 2098 | *0.91* | 5479 | -0.19 | *0.95* |
| Item 10, score 2-3 | -36.0 | 1323 | *0.65* | 1.8 | -127 | 2096 | *0.91* | 4677 | -0.17 | *0.97* |
| Item 11, score 0-1 | -26.2 | 925 | *0.46* | 1.7 | -112 | 1653 | *0.77* | 3221 | -0.21 | *0.98* |
| Item 11, score 1-2 | -33.6 | 1211 | *0.59* | 1.9 | -128 | 2011 | *0.89* | 4696 | -0.19 | *0.97* |
| Item 11, score 2-3 | -35.5 | 1299 | *0.64* | 1.8 | -128 | 2081 | *0.91* | 4519 | -0.17 | *0.97* |
| Item 13, score 0-1 | -27.9 | 978 | *0.46* | 1.9 | -122 | 1779 | *0.79* | 3130 | -0.21 | *0.98* |
| Item 13, score1-2 | -34.5 | 1244 | *0.60* | 1.9 | -131 | 2060 | *0.90* | 4352 | -0.18 | *0.98* |
| Item 13, score 2-3 | -35.8 | 1310 | *0.64* | 1.8 | -127 | 2089 | *0.91* | 5046 | -0.17 | *0.97* |
| Item 14, score 0-1 | -31.5 | 1119 | *0.53* | 1.9 | -128 | 1943 | *0.86* | 4415 | -0.21 | *0.98* |
| Item 14, score1-2 | -35.2 | 1275 | *0.62* | 1.9 | -130 | 2081 | *0.91* | 4957 | -0.19 | *0.96* |
| Item 14, score 2-3 | -35.9 | 1315 | *0.64* | 1.8 | -128 | 2094 | *0.91* | 4779 | -0.17 | *0.97* |
| Item 15, score 0-1 | -32.7 | 1164 | *0.55* | 2.0 | -131 | 2002 | *0.87* | 3953 | -0.19 | *0.99* |
| Item 15, score 1-2 | -35.7 | 1301 | *0.63* | 1.9 | -129 | 2097 | *0.91* | 4875 | -0.18 | *0.96* |
| Item 15, score 2-3 | -36.0 | 1323 | *0.65* | 1.8 | -127 | 2093 | *0.91* | 4680 | -0.17 | *0.96* |
| Item 17, score 0-1 | -34.8 | 1264 | *0.61* | 1.9 | -130 | 2069 | *0.90* | 4277 | -0.17 | *0.98* |
| Item 17, score 1-2 | -35.9 | 1317 | *0.65* | 1.8 | -127 | 2094 | *0.91* | 4423 | -0.16 | *0.98* |
| Item 17, score 2-3 | -36.0 | 1325 | *0.65* | 1.8 | -126 | 2091 | *0.91* | 3794 | -0.15 | *0.98* |
| Item 18, score 0-1 | -30.4 | 1073 | *0.50* | 2.0 | -128 | 1904 | *0.84* | 4041 | -0.22 | *0.99* |
| Item 18, score 1-2 | -35.6 | 1290 | *0.63* | 1.9 | -130 | 2096 | *0.91* | 5164 | -0.19 | *0.98* |
| Item 18, score 2-3 | -36.0 | 1322 | *0.65* | 1.8 | -127 | 2096 | *0.91* | 5207 | -0.18 | *0.96* |
| Item 19, score 0-1 | -32.7 | 1170 | *0.56* | 1.9 | -130 | 1997 | *0.87* | 3899 | -0.19 | *0.99* |
| Item 19, score 1-2 | -35.8 | 1308 | *0.64* | 1.9 | -129 | 2096 | *0.91* | 4819 | -0.17 | *0.97* |
| Item 19, score 2-3 | -36.0 | 1324 | *0.65* | 1.8 | -127 | 2094 | *0.91* | 4477 | -0.16 | *0.96* |
| Item 20, score 0-1 | -29.7 | 1048 | *0.49* | 1.9 | -127 | 1874 | *0.82* | 4251 | -0.23 | *0.98* |
| Item 20, score 1-2 | -35.2 | 1275 | *0.62* | 1.9 | -130 | 2084 | *0.91* | 5222 | -0.19 | *0.96* |
| Item 20, score 2-3 | -35.9 | 1314 | *0.64* | 1.8 | -128 | 2095 | *0.91* | 4558 | -0.17 | *0.97* |
