# Supplementary figures and images for: Boundary curves of individual items in the distribution of total depressive symptom scores approximate an exponential pattern in a general population
Source: PeerJ. 2016 Oct 11;4:e2566. doi: 10.7717/peerj.2566 (PMC5068372; doi:10.7717/peerj.2566)

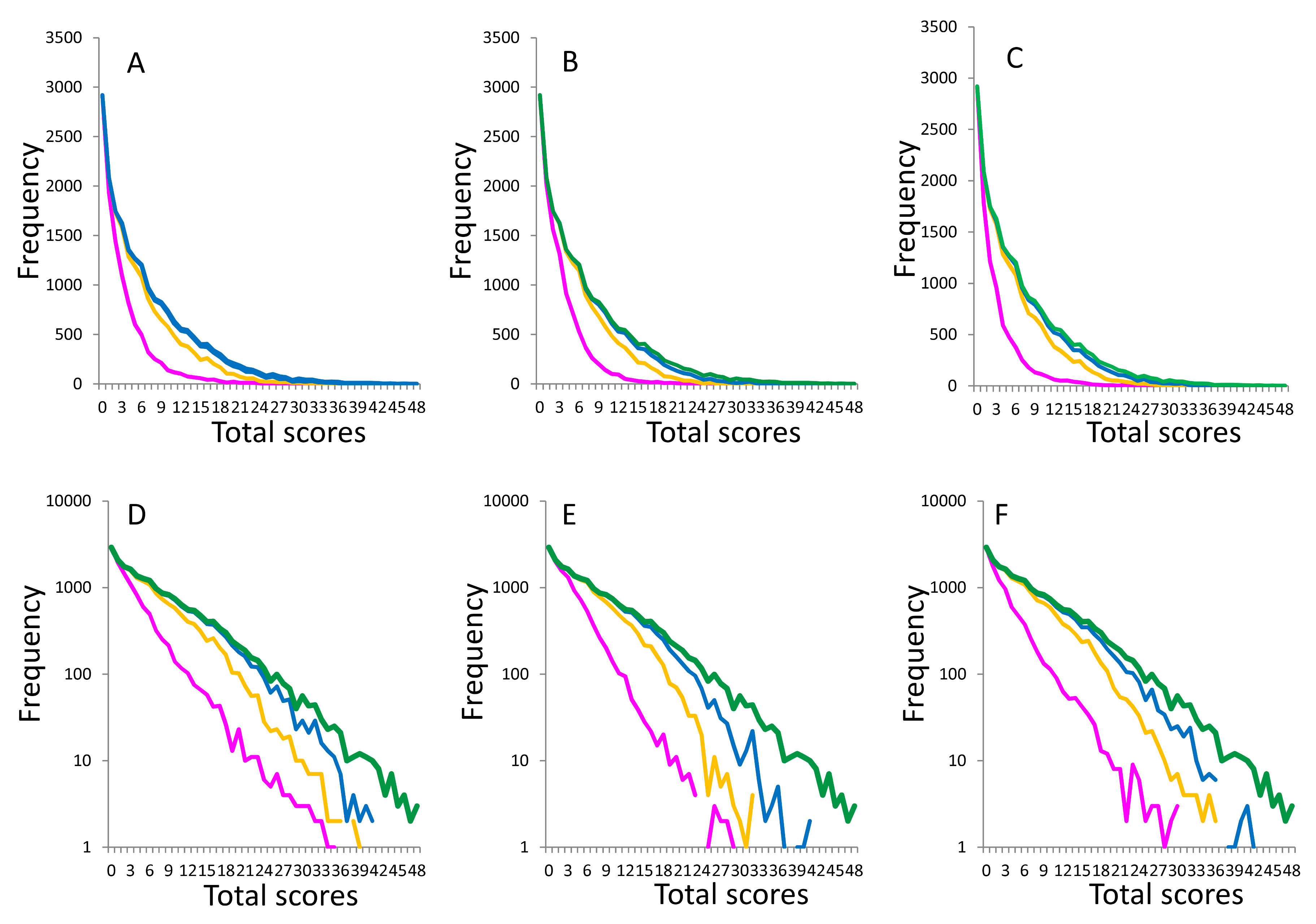

Supplement: Supplemental Information 2 — Boundary curves of item 5 (A, D), item 6 (B, E) and item 7 (C, F) with a normal scale and a log-normal scale are presented, respectively. Magenta, yellow, and blue lines represent the boundary curves between score 0 and score 1, score 1 and score 2, and score 2 and score 3 of Likert scale scores (0-1-2-3), respectively. [file peerj-04-2566-s002.png]

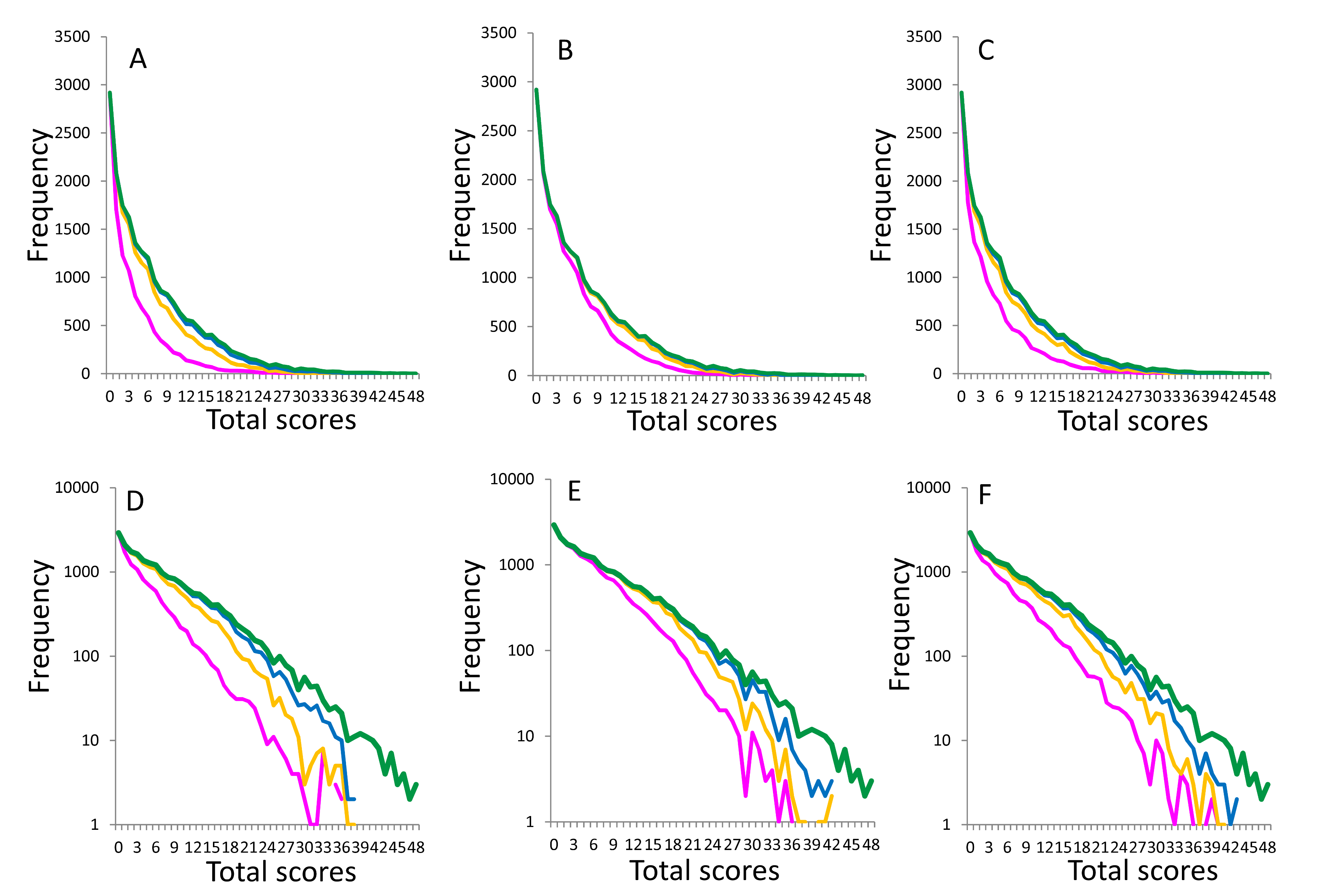

Supplement: Supplemental Information 3 — Boundary curves of item 9 (A, D), item 10 (B, E) and item 11 (C, F) with a normal scale and a log-normal scale are presented, respectively. Magenta, yellow, and blue lines represent the boundary curves between score 0 and score 1, score 1 and score 2, and score 2 and score 3 of Likert scale scores (0-1-2-3), respectively. [file peerj-04-2566-s003.png]

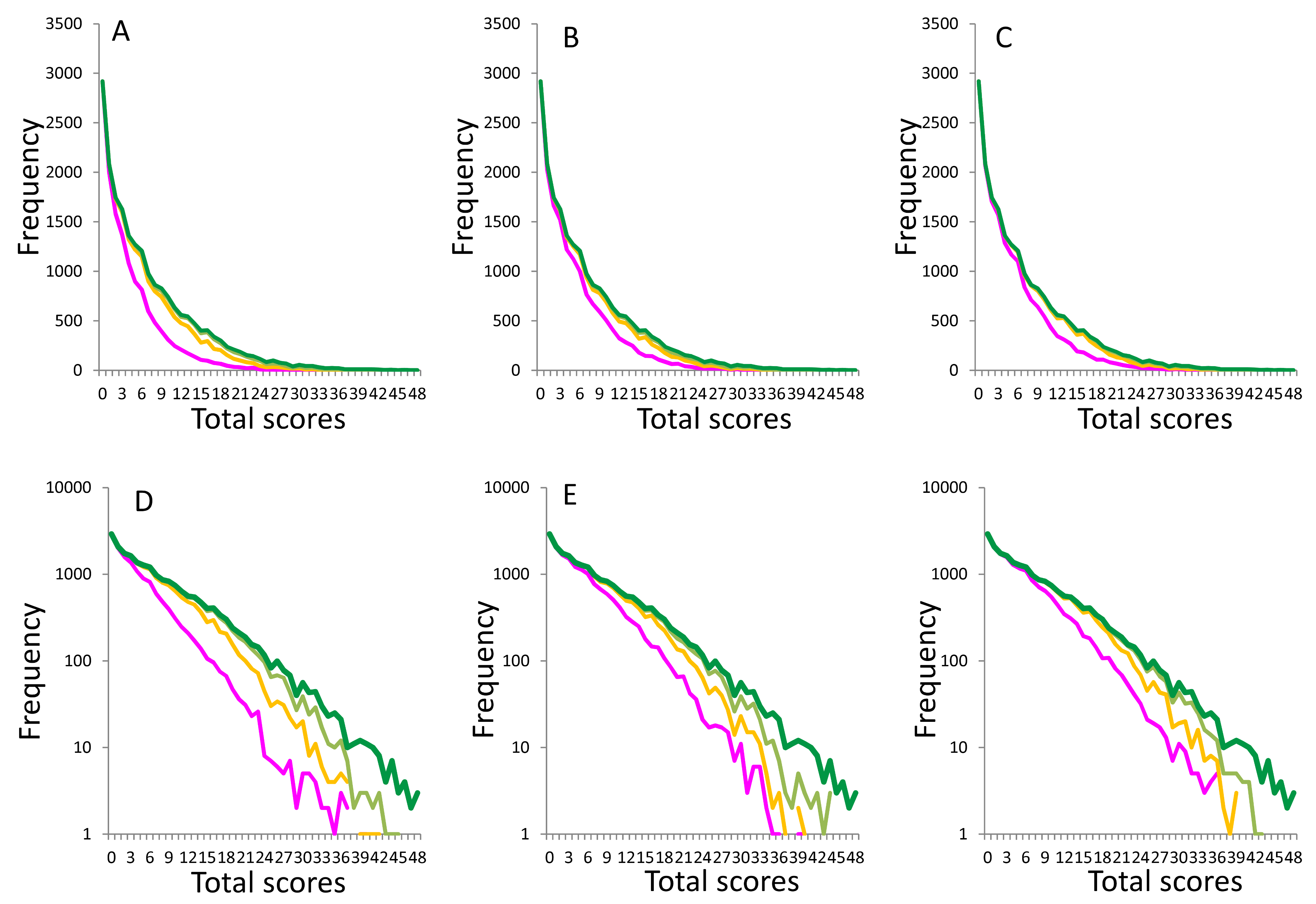

Supplement: Supplemental Information 4 — Boundary curves of item 13 (A, D), item 14 (B, E) and item 15 (C, F) with a normal scale and a log-normal scale are presented, respectively. Magenta, yellow, and blue lines represent the boundary curves between score 0 and score 1, score 1 and score 2, and score 2 and score 3 of Likert scale scores (0-1-2-3), respectively. [file peerj-04-2566-s004.png]

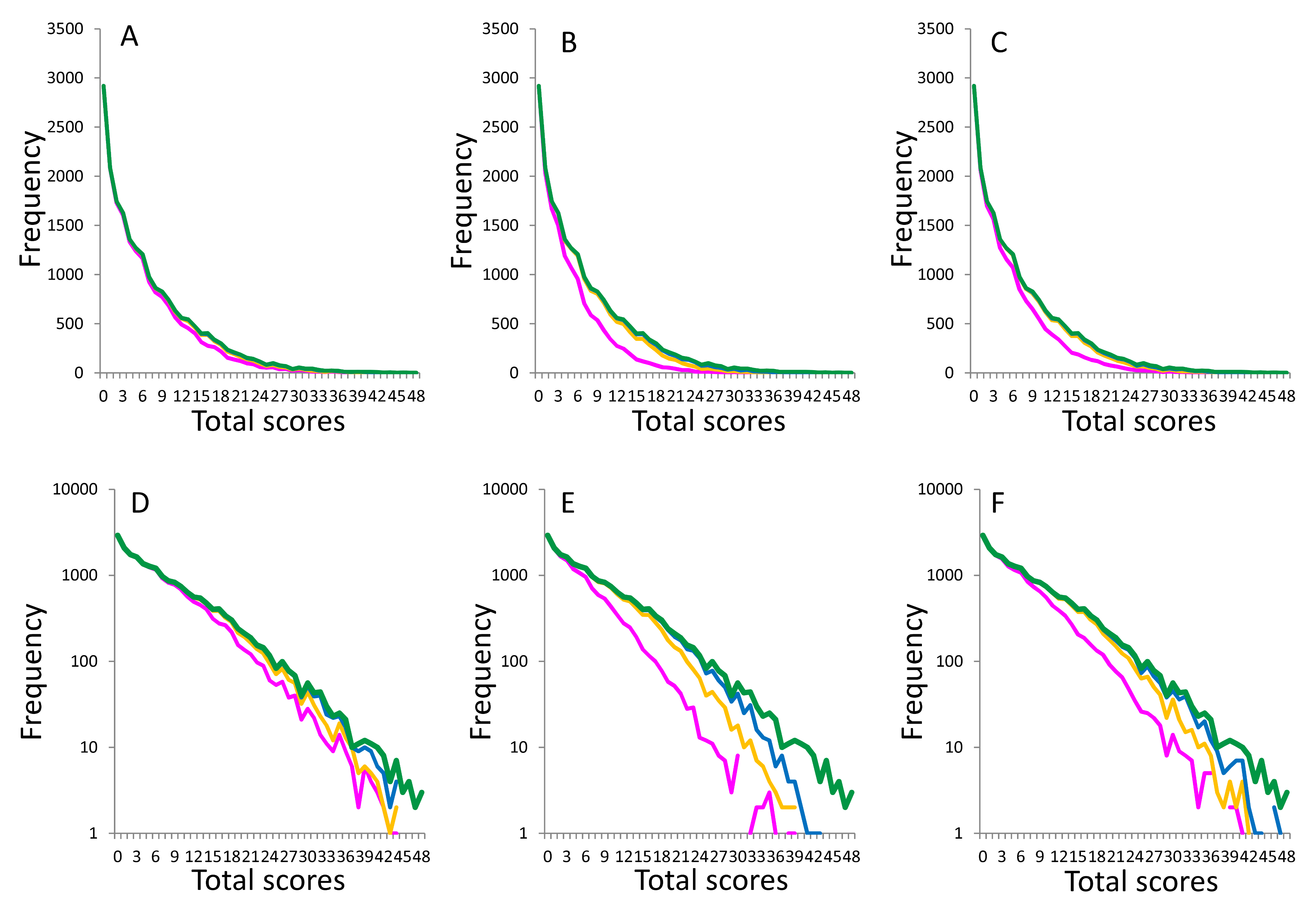

Supplement: Supplemental Information 5 — Boundary curves of item 17 (A, D), item 18 (B, E) and item 19 (C, F) with a normal scale and a log-normal scale are presented, respectively. Magenta, yellow, and blue lines represent the boundary curves between score 0 and score 1, score 1 and score 2, and score 2 and score 3 of Likert scale scores (0-1-2-3), respectively. [file peerj-04-2566-s005.png]

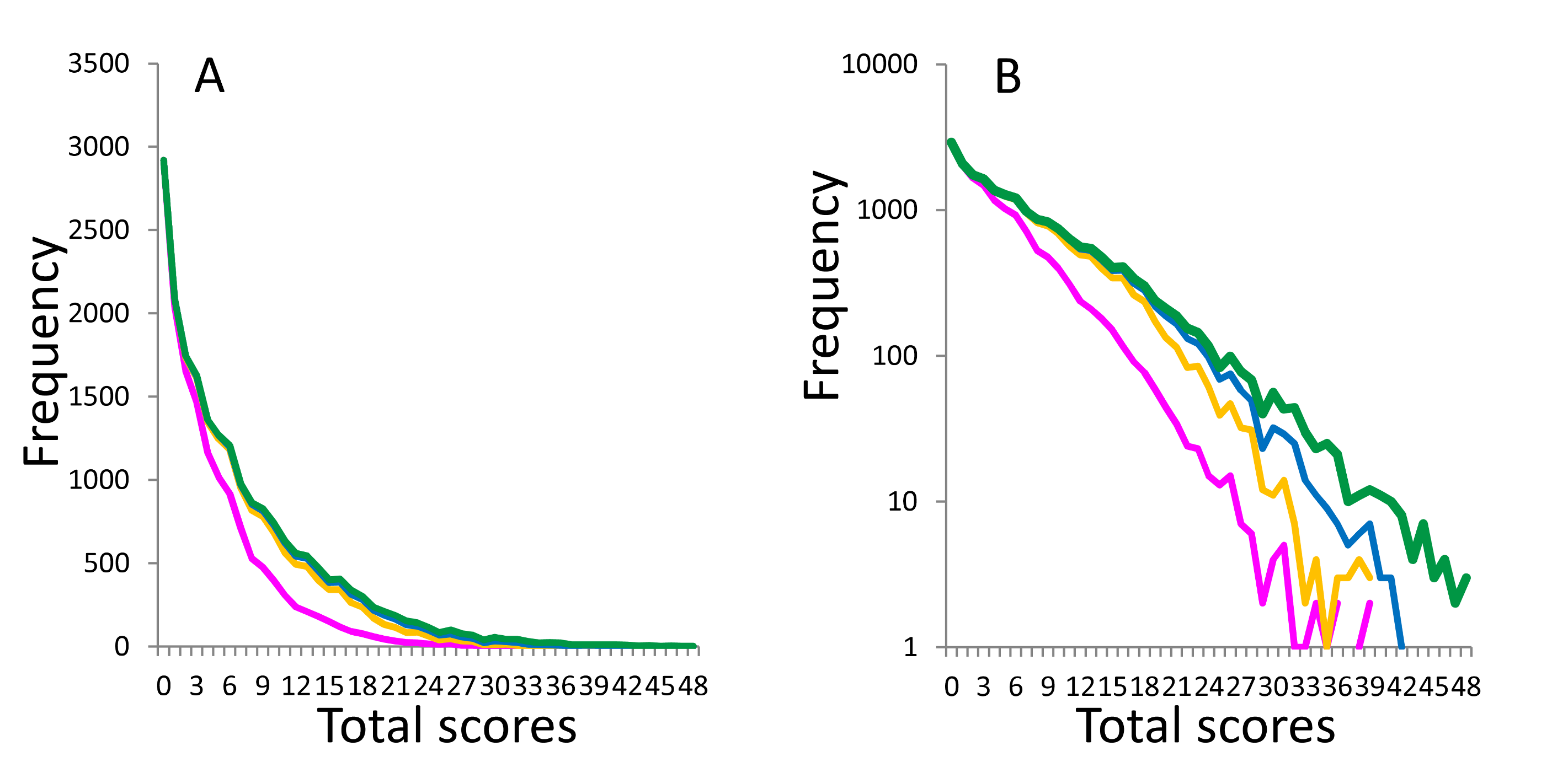

Supplement: Supplemental Information 6 — Boundary curves of item 20 (A, B) with a normal scale and a log-normal scale are presented, respectively. Magenta, yellow, and blue lines represent the boundary curves between score 0 and score 1, score 1 and score 2, and score 2 and score 3 of Likert scale scores (0-1-2-3), respectively. [file peerj-04-2566-s006.png]

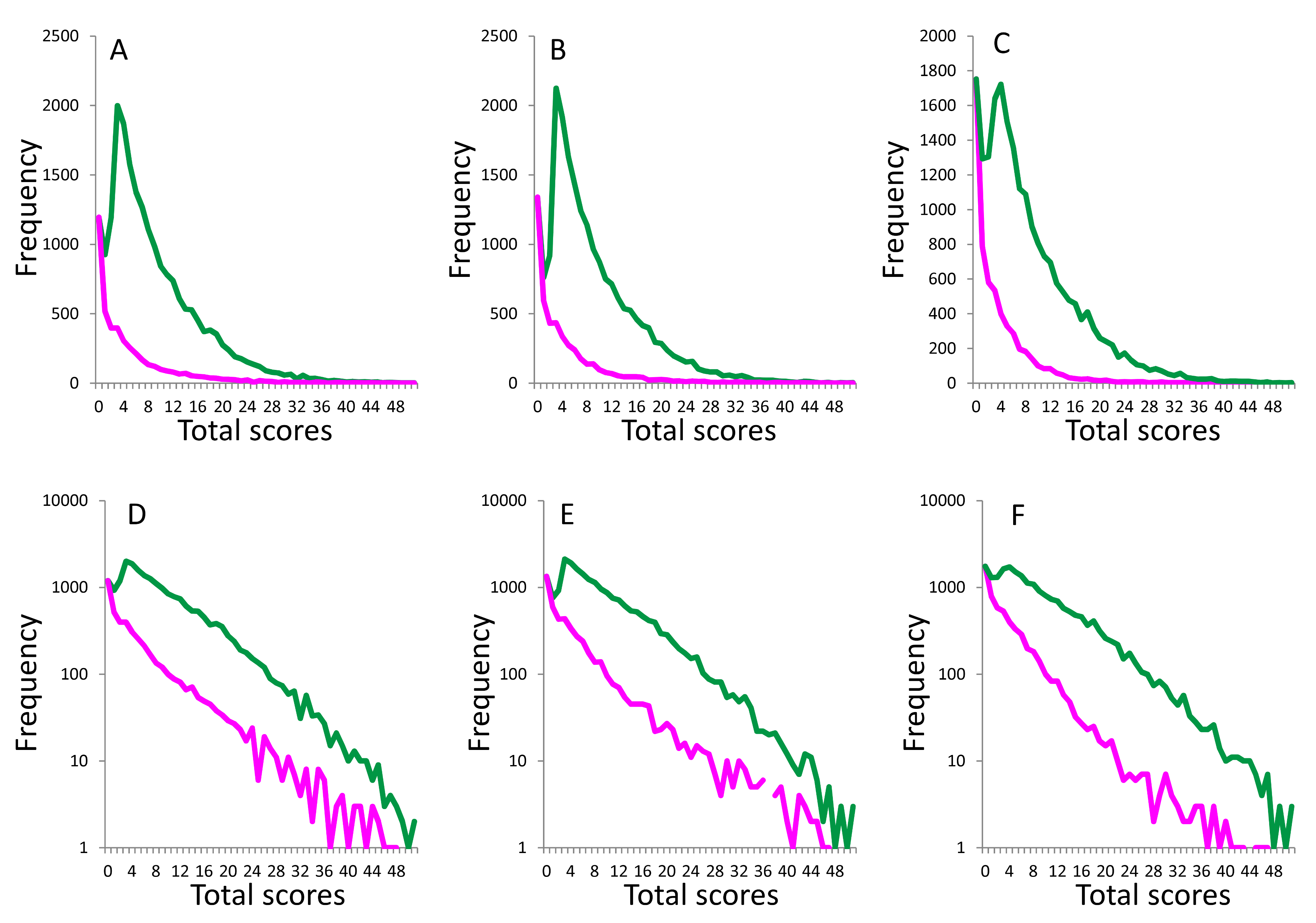

Supplement: Supplemental Information 7 — Boundary curves of item 8 (A, D), item 12 (B, E) and item 16 (C, F) with a normal scale and a log-normal scale are presented, respectively. [file peerj-04-2566-s007.png]
